# Evaluate the developmental competence of human 8-cell embryos by single-cell RNA sequencing

**DOI:** 10.1530/RAF-22-0119

**Published:** 2023-04-19

**Authors:** Weizhou Wang, Mengmeng Zhao, Haiyang Zuo, Jingyao Zhang, Bin Liu, Fu Chen, Pengyun Ji, Guoshi Liu, Shuai Gao, Wei Shang, Lu Zhang

**Affiliations:** 1 Department of Obstetrics and Gynecology, The Sixth Medical Center, Chinese PLA General Hospital, Beijing, China; 2 Department of Obstetrics and Gynecology, Chinese PLA General Hospital, Beijing, China; 3 Department of Animal Reproduction and Development Sciences, College of Animal Science and Technology, China Agricultural University, Beijing, China

**Keywords:** scRNA-seq, zygotic genomic activation, maternal mRNA degradation, 8-cell embryo, developmental competence

## Abstract

**Graphical Abstract:**

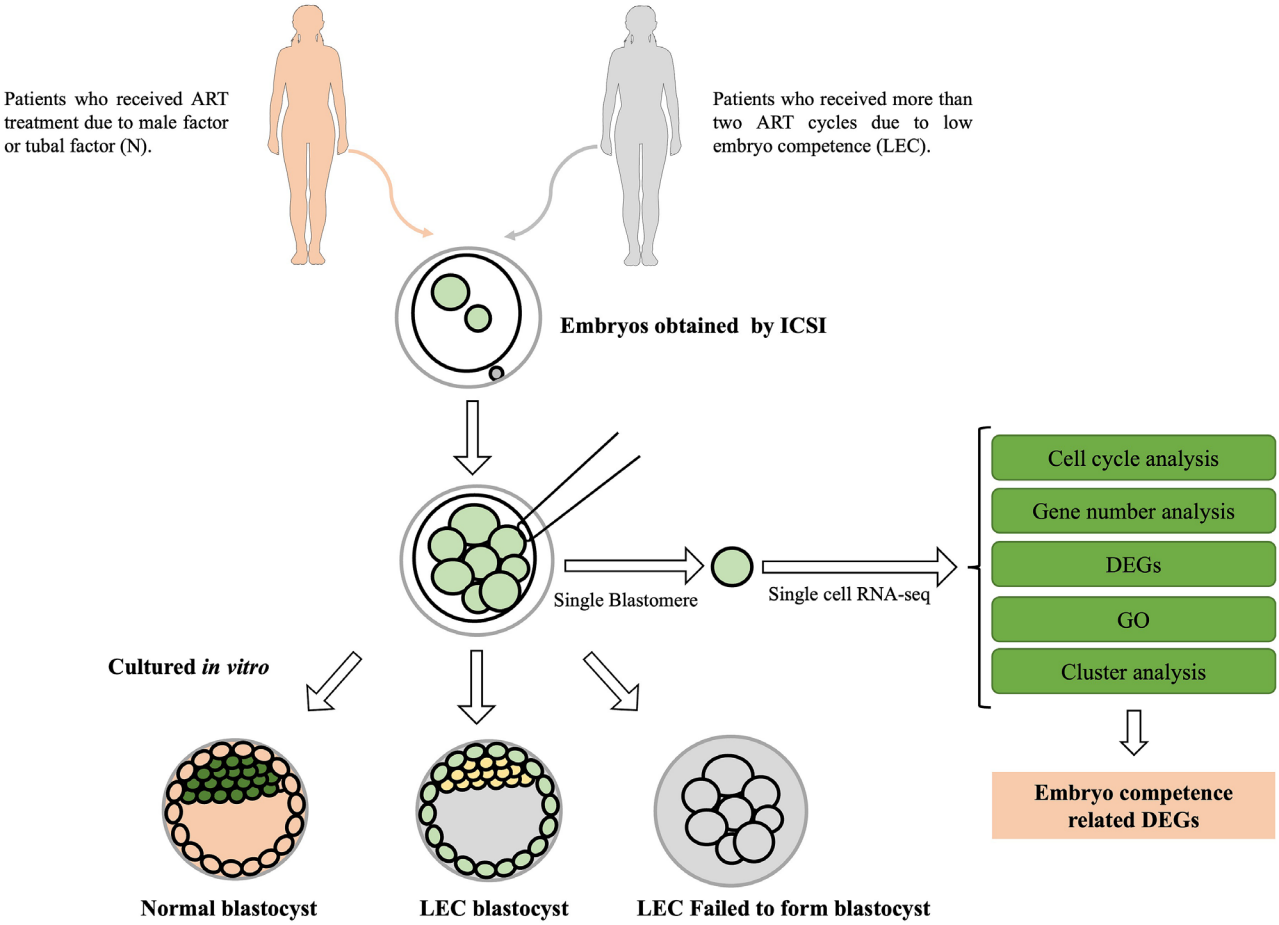

**Abstract:**

The transition of maternal to zygotic gene expression regulation is critical for human preimplantation embryo development. In recent years, single-cell RNA sequencing (scRNA-seq) had been applied to detect the factors that regulate human oocyte maturation and early embryo development. Here, the evaluation of transcriptomes in single blastomere from the embryo collected from patients by scRNA-seq was performed. There were 20 blastomeres biopsied from 8-cell embryos of seven patients who received more than two ART cycles due to low embryo competence. Meanwhile, ten cells were collected from 8-cell embryos of four patients who received ART treatment due to male or tubal factors. The blastomeres were then evaluated using the previously established scRNA-seq method to determine the associations between their gene expression and developmental competence. The total number of genes detected in 8-cell embryos that failed to form blastocyst including maternal and zygotic mRNAs was reduced. There were 324 differently expressed genes detected among the 8-cell embryos including 65 genes that were significantly suppressed in the 8-cell embryos that failed to form blastocyst. Further analysis found these 8-cell embryos arrested at the cleavage stage due to the dysfunction of the cell cycle, DNA transcription activity, histone methylation, and cell division-related genes such as *SMCO-1*,* ZNF271P,ZNF679*,* ASF1b, BEX3, DPPA2*, and* ORC4*. The alterations of gene expression detected in human 8-cell embryos are tightly associated with its developmental competence and could be used as targets to enhance embryo development or parameters to predict the embryo’s development outcomes.

**Lay summary:**

Many females are suffering infertility due to the failure of embryonic development at early stages due to unknown causes. At the very beginning of human embryo development, the embryos start to express its own genes, which should be achieved at 8-cell stage. In current research, we isolated one cell from 8-cell embryos and detected the gene expression at single-cell level. Then the remaining cells of these embryos were cultured to form blastocyst. Meanwhile, the data was analyzed according to the outcomes of embryo development. We detected 324 differently expressed genes between the 8-cell embryos that succeeded and failed to form blastocyst. Our research showed the association between the gene expression and the developmental competence of 8-cell embryos. The findings could be used to predict the embryo quality and potential therapy target to improve the efficiency of assisted reproductive techniques.

## Introduction

The mammalian oocyte deposits a large amount of RNAs and proteins to support the development of preimplantation embryo until the maternal-to-zygotic transition is fully achieved. This process is precisely regulated by a complex of maternal and zygotic factors, and errors in the embryo may cause developmental failure or long-term outcomes (Schulz & Harrison 2019). Nowadays, assisted reproductive techniques (ARTs) have helped millions of couples to have their own child. The ART is still not efficient enough to reduce the sufferings of patients who experienced embryo development failure due to the limited knowledge about human embryo development. During maternal-to-zygotic transition process, early embryos are highly sensitive to change its surrounding conditions such as maternal health and diet, *in vitro* embryo culture medium, temperatures, and O level (El Hajj & Haaf 2013). With the advanced research methods at the molecular level, we now have the opportunity to elucidate the gene expression changes that determine embryo competence.

Following fertilization, the activation of the zygote genome is initiated and maternal mRNAs are eliminated (Clift & Schuh 2013). The research on human, mouse, goat, and bovine has demonstrated that the elimination of maternal transcripts is accomplished by two sequential pathways mediated by maternal or zygotic factors (Xue *et al.* 2013, Yan *et al.* 2013, Bogliotti *et al.* 2020, Deng *et al.* 2020, Sha *et al.* 2020*a*). Recent studies using single-cell RNA sequencing (scRNA-seq) found that about 78.94% of 7271 genes (human) or 57.49% of 8081 (mouse) maternal transcripts were degraded in 8-cell stage human embryos or 2-cell mouse embryos (Sha *et al.* 2020*a*). The functional studies already reported that genes such as *MYS2, PABPN1L, YTHDF2*, and *BTG4* are critical for mRNA clearance in human and mouse embryos (Yu *et al.* 2001, Ivanova *et al.* 2017, Sha *et al.* 2020*b*, Zhao *et al.* 2020). The dysfunction of the above genes causes embryo development failure.

Another essential event in the maternal-to-zygotic transition is zygotic genome activation (ZGA). For the human embryo, the initiation of ZGA begins in the zygote, and the major ZGA occurs around the 8-cell stage (Dobson *et al.* 2004, Asami *et al.* 2022, Du *et al.* 2022). Many transcription factors that regulate human genome activation such as *OCT4, DUX4,* and *HERVL* have been identified using low-input sequencing methods (Choi *et al.* 2016, Fogarty *et al.* 2017, Hendrickson *et al.* 2017). The disruptions in ZGA are often lethal during embryogenesis or cause long-term developmental defects and diseases (Greenberg & Bourc'his 2019, He *et al.* 2022). But the whole picture of maternal-to-zygotic transition is not fully elucidated yet.

Over the past decade, a significant advance in sequencing techniques has been made, and the costs of sequencing have also been dramatically reduced. Nowadays, scRNA-seq has become an alternative approach to evaluating the transcriptome in the human early embryos. Here, we applied scRNA-seq to investigate the potential factors in 8-cell embryos that affect the development of the human embryo. Current research provided evidence that the dysfunction of mRNA clearance or zygotic gene transcription affects human preimplantation embryo development. In the clinical investigation, when preimplantation genetic diagnosis (PGD) is performed, the evaluation of transcriptions could be used as a new parameter to predict the embryo developmental potential.

## Materials and methods

### Ethics statement

All procedures involving human participants were performed in accordance with the ethical standards approved by the Medical Ethics Committee of the Sixth Medical Center of Chinese PLA General Hospital (Reference No. HZKY-PJ-2021-34) and with the Declaration of Helsinki 1964 and its later amendments. A total of 11 female donors aged 28–40 years were recruited, and all the donor couples were informed with written consent, confirming that the voluntarily donated embryos were used only for scientific research on human early embryonic developmental mechanisms.

### Ovarian stimulation, fertilization, and embryo culture

The ovarian stimulation of the donors was performed according to clinical protocols, and vum pickup was carried out 36 h later through vaginal puncture under ultrasound guidance, following standard clinical procedures at the Reproductive Medical Center of the Sixth Medical Center of Chinese PLA General Hospital (Qi *et al.* 2022). Oocytes were inseminated through intracytoplasmic sperm injectoin (ICSI) using a micromanipulation system (Nikon Instruments, Tokyo, Japan). Each embryo was incubated in a separate G1 medium (Vitrolife, Vastra Frolunda, Sweden) droplet overlaid with paraffin oil, and then placed in the incubator (ThermoFisher, MA, USA) with the setting of 37℃, 5% CO_2,_ and 100% humidity inside, until the biopsy on Day 3.

### Blastomere biopsy for scRNA-seq

**Figure 1:**
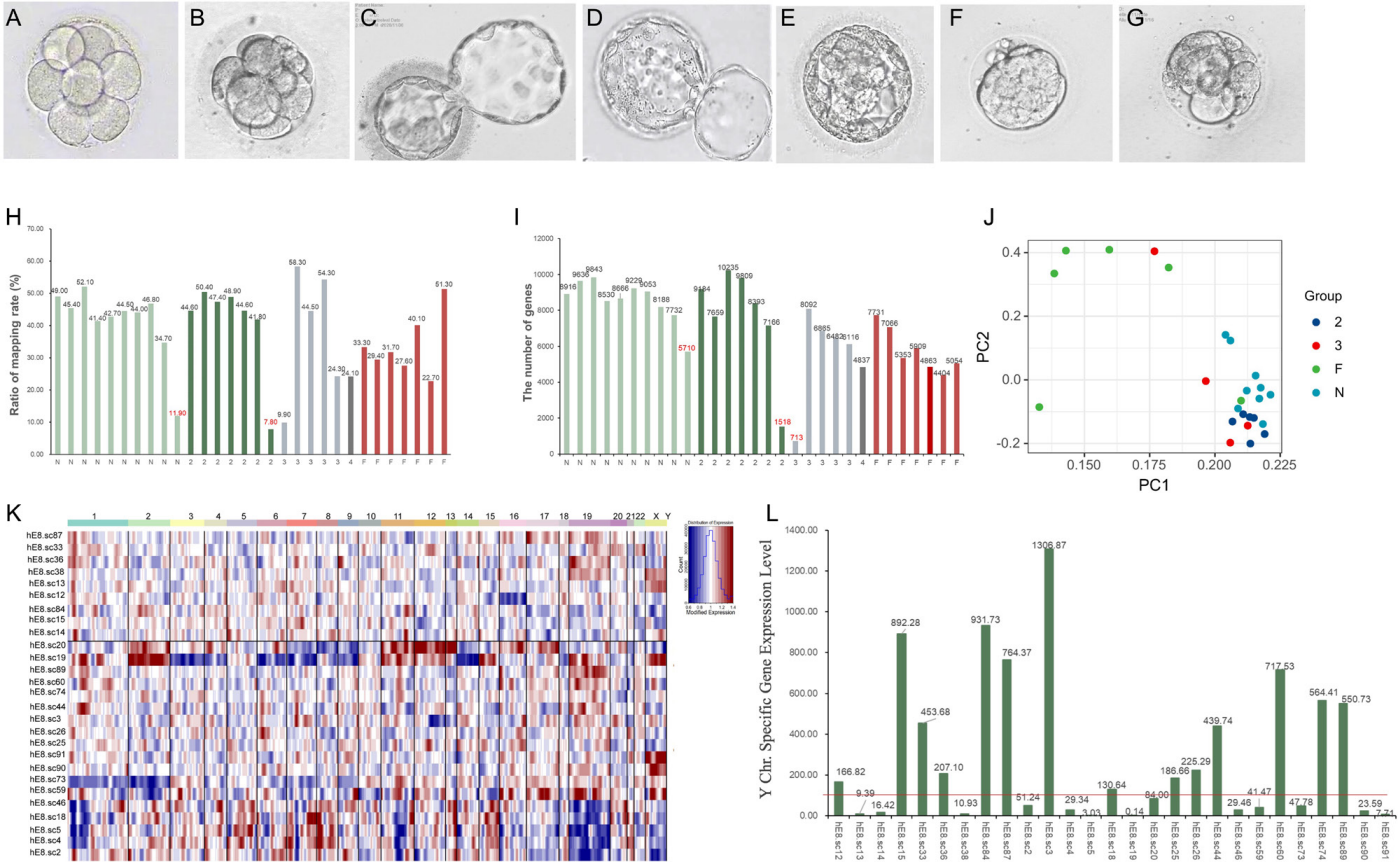
The development of biopsied embryos and scRNA-seq data processing. (A&B) Representative images of 8-cell embryos. (C-G) Representative images of Grade 1, 2, 3, and 4 blastocyst and embryos that failed to form blastocyst. (H) The histogram of genomic mapping rate for each sample (the samples with genomic mapping rate < 20% were marked as red). (I) The histogram of the number of genes detected in each sample (the samples with gene number < 4000 were colored as red). (J) Principal component analysis of the expression patterns from scRNA-seq data among embryos with different developmental outcome indicated by colored dots. (K) The heatmap of the gene expression levels in different chromosomes calculated by inferCNV package based on the average expressions of 50 upstream and downstream genes according to their genomic locations (CNV values by chromosome showed in columns for individual samples (rows)). (L) The expression levels of Y chromosome-specific genes of all embryos (the samples with the sum of chromosome-Y (chrY) RPKM (*∑RPKM_chrY_*) >100 were inferred as male and samples with* ∑RPKM_chrY_* <50 were classified as female).

On day 3, the blastomere biopsy for scRNA-seq was performed 67–73 h after ICSI. Only when the patient had more than five embryos graded as 1–3 at the 8-cell stage, they embryos were selected (Fig. 1A and B). Prior to biopsy, the embryos were incubated in Ca^2+^/Mg^2+^ free medium for 2–5 min. A ~30 μm opening in the zona pellucida was generated by laser (Hamilton Thorne Research, MA, USA), and one blastomere for each embryo was biopsied. The single blastomere was then transferred into cold scRNA-seq lysis buffer respectively using a mouth pipette. The remaining cells of the embryos were cultured in G2 medium (Vitrolife, Vastra Frolunda, Sweden) and were further cultured up to Day 6 until blastocyst assessment.

### Morphology assessment and grading of the blastocysts

The morphology of the blastocysts was evaluated according to Gardner's scoring system (Gardner *et al.* 2000). Briefly, a grade was given according to the degree of blastocyst expansion and hatching status, and the quality of inner cell mass (ICM) and trophectoderm (TE). As a summary, we presented the grade in current manuscript as Grades 1–4 and failed to form blastocyst (F).

### scRNA-seq library construction, sequencing, and data preprocessing

The protocol for scRNA-seq library preparation and sequencing was modified according to previous research (Gao *et al.* 2018). The libraries were sequenced with Illumina HiSeq 4000 (Illumina, CA, USA). Single-end sequencing reads were initially split according to the cell-specific barcodes. After the TSO or poly (A) sequence was removed from the reads, quality control was performed to eliminate adapter contamination and low-quality bases. Next, the cleaned sequencing reads were mapped to the human genome (hg19) using Hisat (v2.2.1), and only uniquely mapped reads were kept (Kim *et al.* 2015). Then the numbers of the reads mapped to each gene were counted using StringTie v2.1.7 (Pertea *et al.* 2015).

### Copy number variation, embryonic sex, and cell cycle analysis

The copy number variation (CNV) assessment for each embryo was performed using the inferCNV package (v1.6.0). Briefly, the normalized gene expression values were calculated based on the average expressions of 50 upstream and downstream genes according to their genomic locations, following the method reported in the previous researches (He *et al.* 2022). The sex of each embryo was determined according to the expression of Y-linked genes as in previous researches (Petropoulos *et al.* 2016). As described in a previous study (Li *et al.* 2017), we next analyzed the expression levels of cell cycle regulation-related genes, including the core set of G1/S and G2/M related genes based on the average fragments per kb per million mapped fragments (FPKM) and the log_2_(FPKM+1) value.

### The analysis of the differentially expressed genes

The expression of genes was normalized with FPKM, and all data were normalized for further analysis. The differential expression genes (DEGs) between groups were determined by the DESeq2 package (Love *et al.* 2014). A gene was considered significant if the adjusted *P* value (*Padj*.) was<0.05 and the |log_2_ (Fold change) | ≥ 1. The maternal mRNAs were analyzed using previously published dataset (Xue *et al.* 2013).

We applied the fuzzy c-means algorithm in the Mfuzz R package (version 2.52.0) to profile all DEGs according to their expression patterns, and each gene was assigned a unique cluster according to its value calculated by clusterProfiler v4.0.5 (Kumar & Futschik 2007, Yu *et al.* 2012). Gene Ontology (GO) pathways enrichment analyses of DEGs were performed (Ogata *et al.* 1999). And the correlation between gene expression and embryo quality was analyzed by Pearson correlation coefficient values of pairwise comparisons. Finally, the expression levels of the DEGs that expressed in human preimplantation embryo as shown in DevOmics database (http://www.devomics.cn) (Yan *et al.* 2021), or involved in embryo development reported in previous literature, were compared for different developmental outcomes.

### Statistical analysis

All data were analyzed by R or GraphPad Prism 9.0.0. All experiments included at least three independent samples. Statistical data were presented as mean ± s.e.m., and one-way ANOVA was performed to determine the significance. Statistically significant values of *P <* 0.05, *P <* 0.01, and *P <* 0.001 by two-tailed Student’s *t*-test are indicated by asterisks (*), (**), and (***), respectively. And ‘ns’ indicates that the difference is non-significant.

## Results

### The development of biopsied embryos and scRNA-seq data processing

There were 20 single blastomeres biopsied from seven patients who received more than two ART cycles due to low embryo competence (LEC). Twelve of the 20 embryos (60.00%) developed into good-quality embryos (Grade 1–3, Fig. 1C, D and E), and eight of them failed to form blastocysts (Fig. 1F and G). Meanwhile, ten cells biopsied from 8-cell embryos from four patients who received ART treatment due to male factor or tubal factor were assigned as N group, and all these 8-cell embryos developed to Grade-2 or Grade-3 (10/10) blastocysts (shown in Table 1).

**Table 1:** The biopsy and development of 8-cell embryos and scRNA-seq data processing. Data are presented as *n* or *n* (%).

	LEC	Normal
8-cell embryos, *n*	20	10
Grade 2	7 (35%)	8 (80%)
Grade 3	5 (25%)	2 (20%)
Grade 4	1 (5%)	0 (0%)
Grade F	7 (35%)	0 (0%)
scRNA-seq data qualified	18	9
Male/female	9/11	6/4

Subsequently, the scRNA-seq data was processed, and the blastomeres with poor RNA quality were filtered out (genomic mapping rate < 20% or gene number < 4000, shown in Fig. 1H and I and listed in Supplementary Table 1. see section on supplementary materials given at the end of this article). The data from 18 embryos in LEC group and 9 embryos in N group were used for following analysis. The principal component analysis (PCA) of scRNA-seq data was performed using an unsupervised approach, and the 27 blastomeres could be divided into two subgroups (Fig. 1J), suggesting the heterogeneity that existed among the embryos. Thus, the scRNA-seq data from one single blastomere was analyzed to represent the transcriptional status in the whole embryo.

The copy number variation (CNV) of the chromosomes was also analyzed to determine the embryonic aneuploidy. As shown in Fig. 1K, two 8-cell embryos that developed to Grade 4 or F blastocysts showed partial deletions on chromosome 1, 2, 3, 14, and 16 (blue) or chromosome two gain (red). Data obtained from embryos were not used for further analysis. The embryonic sex of all samples was also inferred by analyzing the expression level of specific gene on Y chromosome (Fig. 1L and Table 1). It was found that four embryos were female and the other six were male in the N group, and all the embryos developed to the blastocyst stage. In the LEC group, four of the eleven female embryos developed into blastocysts and seven of the nine male embryos formed blastocysts.

### The differences of gene number among the 8-cell embryos

**Figure 2:**
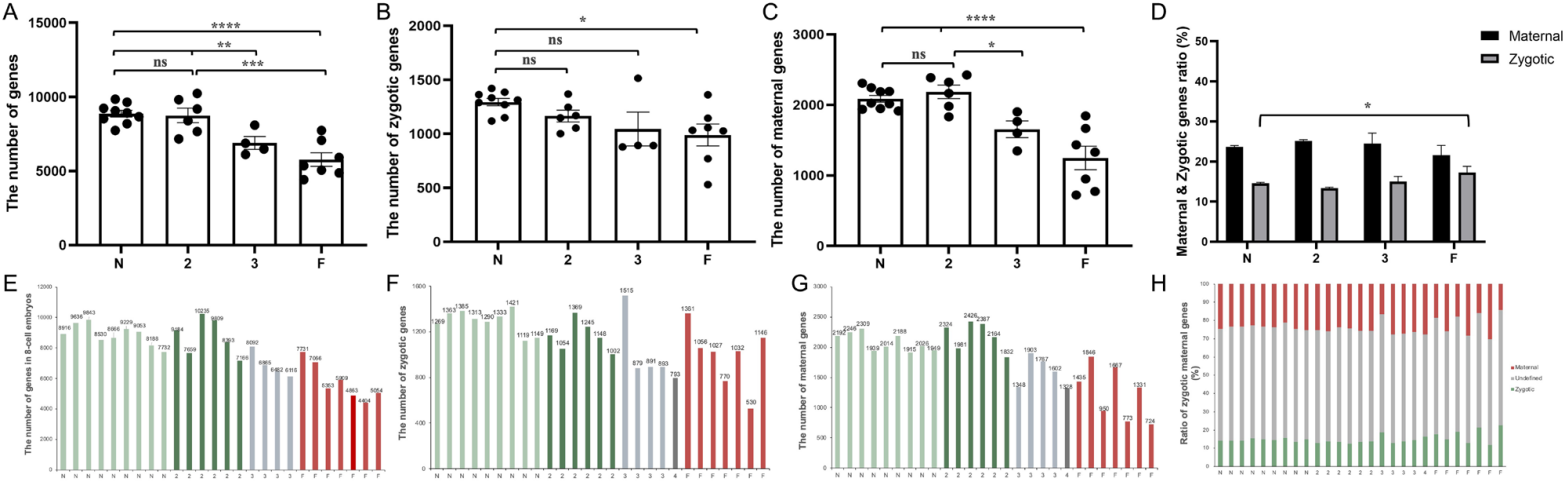
The gene number differences between the high- and low-quality 8-cell embryos. (A-C) The histogram of the general gene (A), zygotic genes (B), and maternal (C) genes number detected in single blastomere of 8-cell embryos. Data are presented as mean values ± s.e.m. *P* by one-way ANOVA. ns, non-significant. * *P* < 0.05, **** *P <* 0.001. (D) The ratio of maternal and zygotic genes. Data are presented as mean values ± s.e.m., and *P*was calculated using one-way ANOVA. ns non-significant. * *P* < 0.05. (E-G) The histogram shows the number of general genes (E), zygotic genes (F), and maternal (G) genes in each sample. (Different column colors represent different embryo class.) (H) The ratio of maternal and zygotic genes for each sample.

The gene number detected in the blastomere could indicate the transcriptional active level in preimplantation embryos. As shown in Fig. 2A (listed in Fig. 2E), the number of genes detected in the 8-cell embryos that failed to form blastocyst (5769 ± 460.8) and that formed Grade 3 blastocyst (6889 ± 429.2) was significantly lower than that in 8-cell embryos that developed to Grade 2 (8741 ± 494.7) and N group (8866 ± 224.1) blastocysts. Further analysis determined that the expression of both the zygotic and maternal genes in the F embryos (988.9 ± 101.1 and 1247 ± 166.5) was significantly reduced than that in the N group (1294 ± 34.02 and 2086 ± 49.35) (Fig. 2B and C). Similarly, there were more maternal mRNAs detected in the 8-cell embryos that formed Grade 2 blastocyst (2186 ± 97.45) than that in Grade 3 (1655 ± 119.4) and F embryos. It is noticeable in Fig. 2D and H that the percentage of zygotic genes detected in 8-cell embryos that failed to form blastocysts (17.24 ± 4.10%) was higher than that from Grade 2 embryos (13.37 ± 0.55%).

### The expression of cell cycle regulation related genes in 8-cell embryos

**Figure 3:**
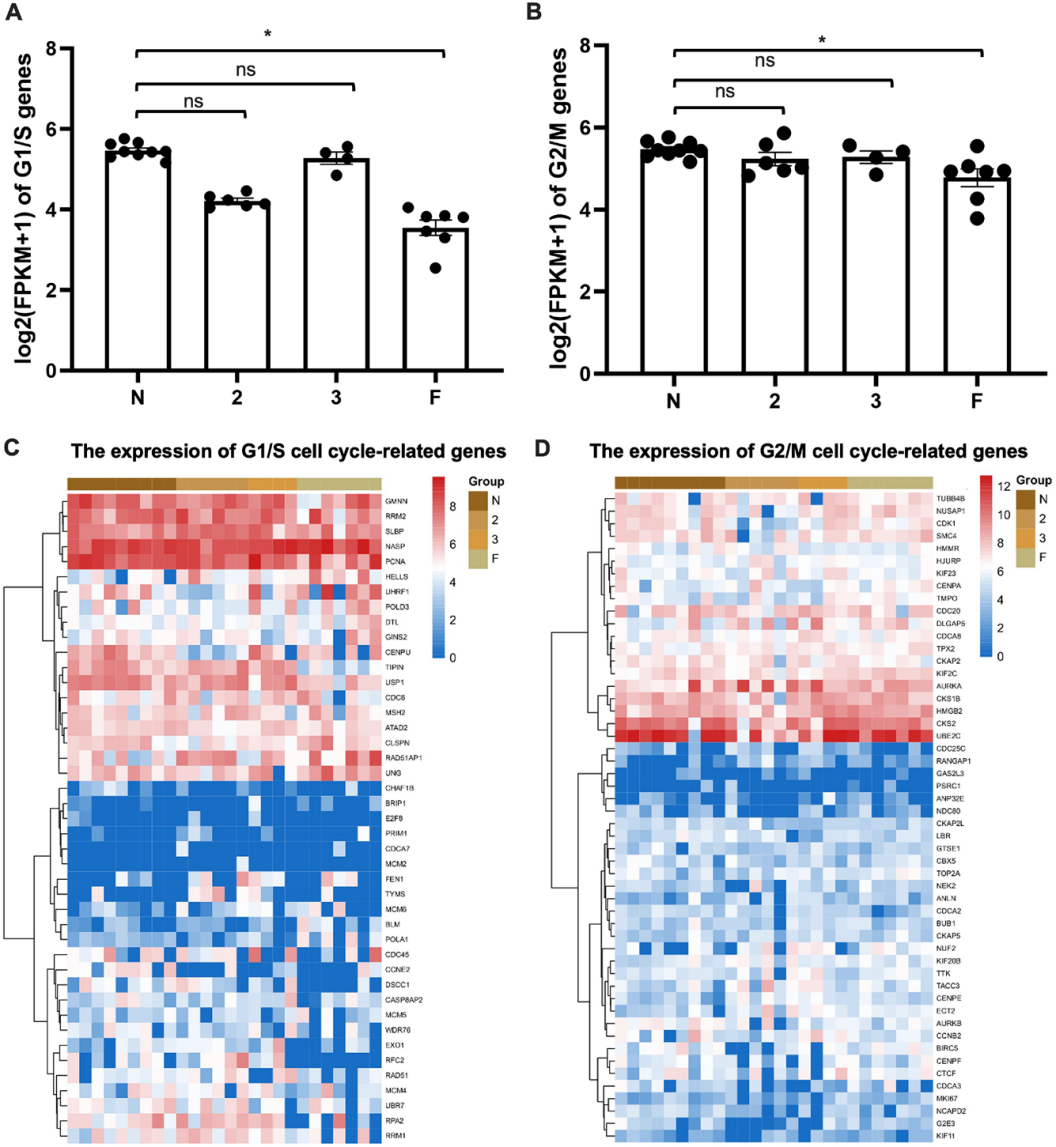
The difference of cell cycle regulation genes between the high- and low-quality 8-cell embryos. (A) The average log2 (FPKM+1) of G1/S cell cycle-related genes in the 8-cell embryos of each group. (B) The average log2 (FPKM+1) of G2/M cell cycle-related genes in the 8-cell embryos of each group. Data are presented as mean values ± s.e.m. *P* value was calculated using one-way ANOVA. ns non-significant. * *P* < 0.05. (C and D) The expression level of cell cycle-related genes among the 8-cell embryos of each group. The color keys from blue to red indicate the relative gene expression levels from low to high.

The genes that participated in cell cycle regulation were investigated in each embryo according to the methods described in previously published articles (Li *et al.* 2017). The average expression of both G1/S and G2/M related genes in Fig. 3A (listed in Fig. 3C) in the 8-cell embryos that failed to form blastocyst was significantly lower than that in the N group embryos. It was noticeable that G1/S related genes in Grade 3 embryos were more active than that in F embryos. The detailed expression level of cell-cycle related genes is presented in Fig. 3B and D. The current result demonstrated that there was obvious difference in the expression of cell cycle-related genes between the low- and high-quality 8-cell embryos, which coordinated with the fast cleavage stage of preimplantation embryo.

### The differences of gene expression between the 8-cell embryos

**Figure 4:**
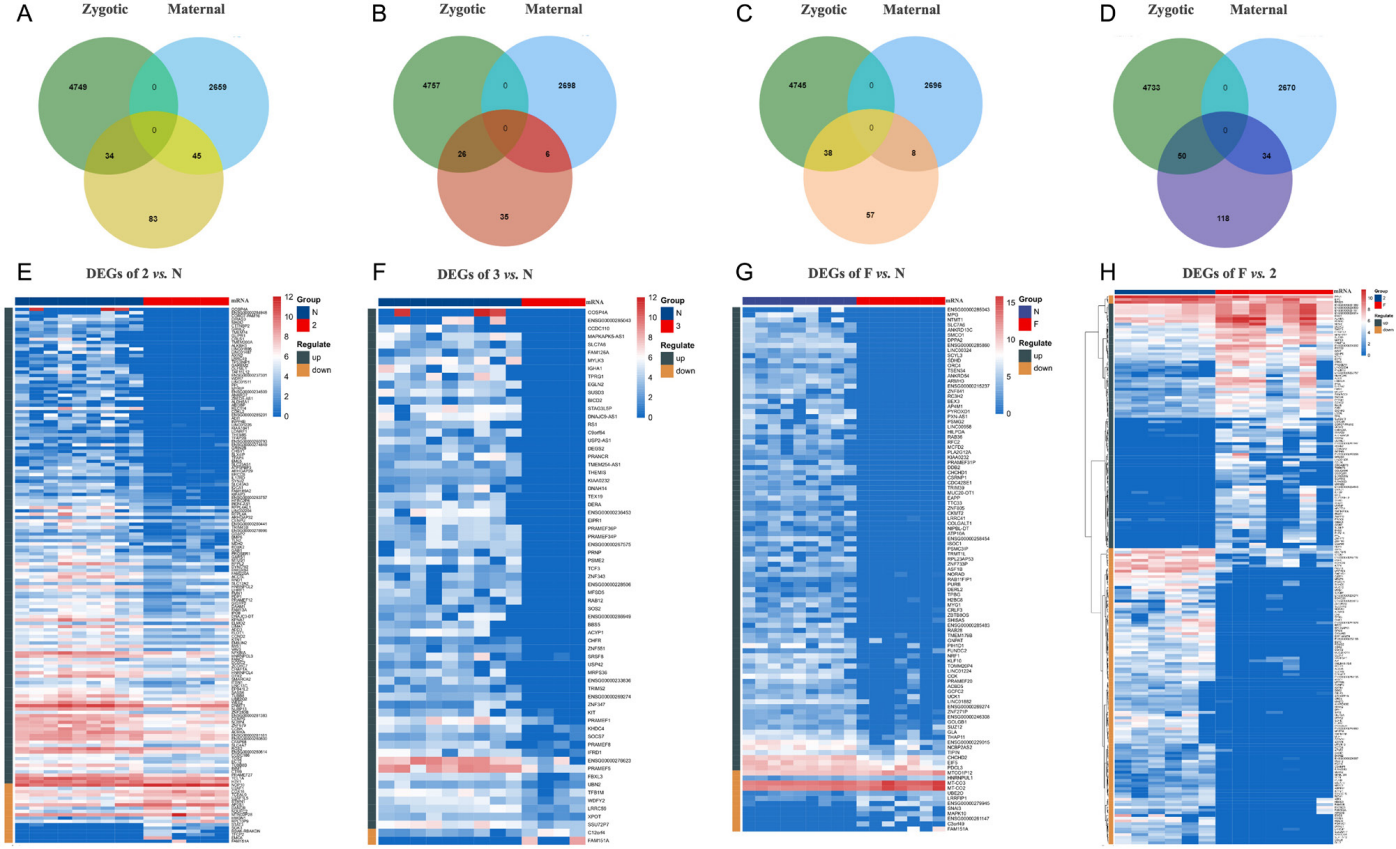
The differences of gene expression between 8-cell embryos. (A-D) Venn Diagram to show the number of zygotic and maternal mRNAs in each comparison pair. (E-H) The heatmap of detailed DEGs in each comparison pair. The color keys from blue to red indicate the relative gene expression levels from low to high.

The differences of gene expression (DEGs) between the 8-cell embryos that showed high and low developmental competence were analyzed with DESeq2 (Fig. 4 and Supplementary Table 2). Compared with N embryos, there were 162 DEGs detected between Grade 2 embryos (Fig. 4A and E) and 67 DEGs in the Grade 3 embryos (Fig. 4B and F). To further explore the reasons behind human blastocyst formation failure, we characterized the 103 DEGs between F and N embryos, including 38 zygotic genes (36 upregulated and 2 downregulated) and eight maternal genes (5 upregulated and 3 downregulated) (Fig. 4C and G). There were 202 DEGs that were identified between Grade 2 and F embryos, and 112 genes (42 zygotic and 4 maternal) were reduced and 90 genes (8 zygotic and 40 maternal) were elevated (Fig. 4D and H).

**Figure 5:**
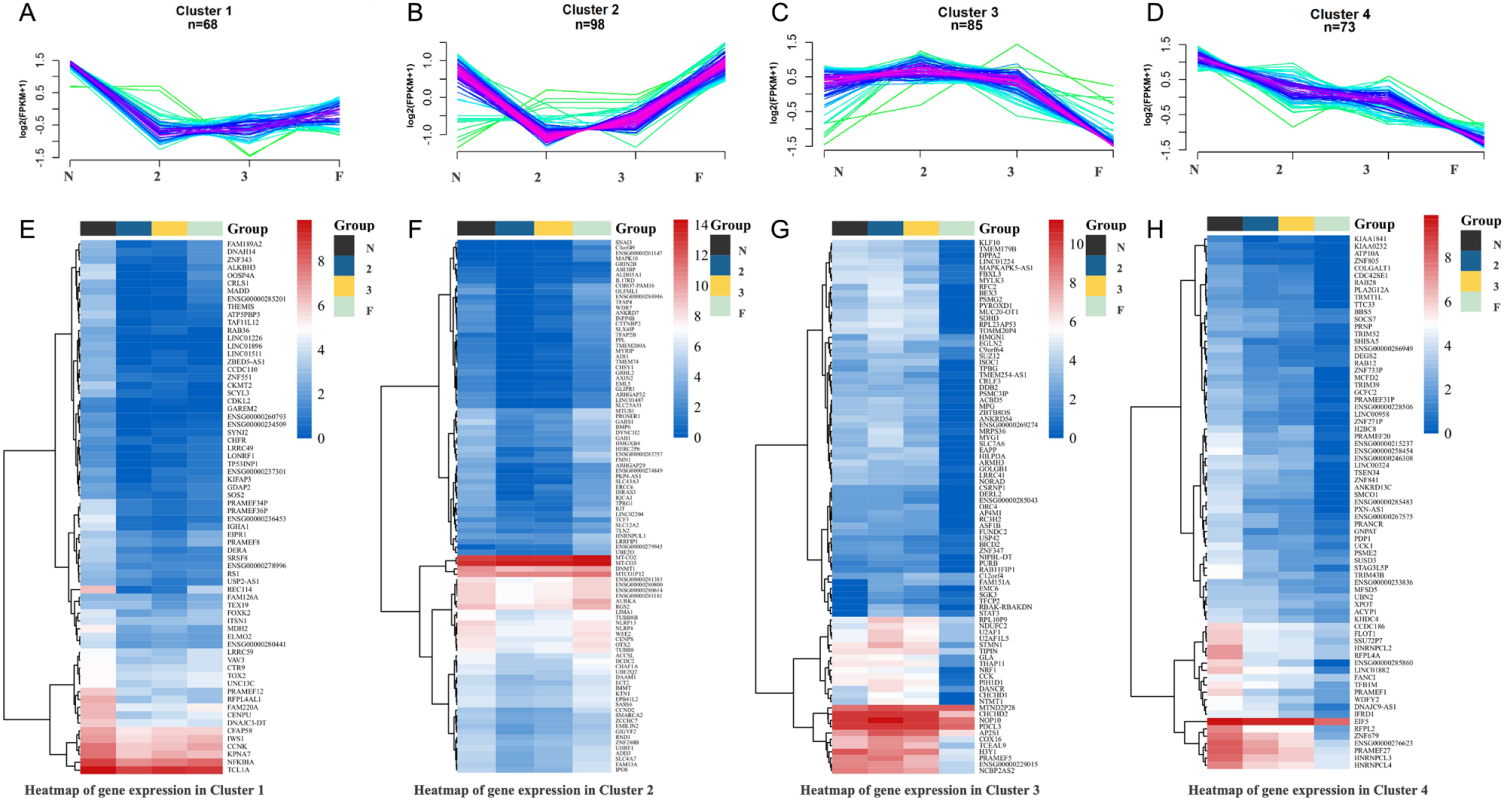
The patterns of the differently expressed genes in 8-cell embryos. (A-D) Expression pattern of all DEGs. Each line shows the average expression level of one gene from all samples. (E-H) Heatmap representing gene mean FPKM expression in each cluster. The color keys from blue to red indicate the relative gene expression levels from low to high.

The patterns of all the 324 DEGs were evaluated by series test of cluster, which were classified into four clusters (Fig. 5 and Supplementary Table 3). Cluster Ⅰ (Fig. 5 A and E): the expression level of 68 genes was lower in all the 8-cell embryos collected from patients with low embryo quality than that from the N group. Cluster Ⅱ (Fig. 5B and F): there were 98 genes showing lower level in Grade 2, 3 than that in F and N. Cluster Ⅲ (Fig. 5C and G): the expression of 85 genes was suppressed in 8-cell embryos that failed to form blastocysts than other embryos. Cluster Ⅳ (Fig. 5D and H): the expression of 73 genes was continually reduced from N to 8-cell embryos that failed to form blastocyst.

### Correlation between gene expression and embryo developmental competence

**Figure 6:**
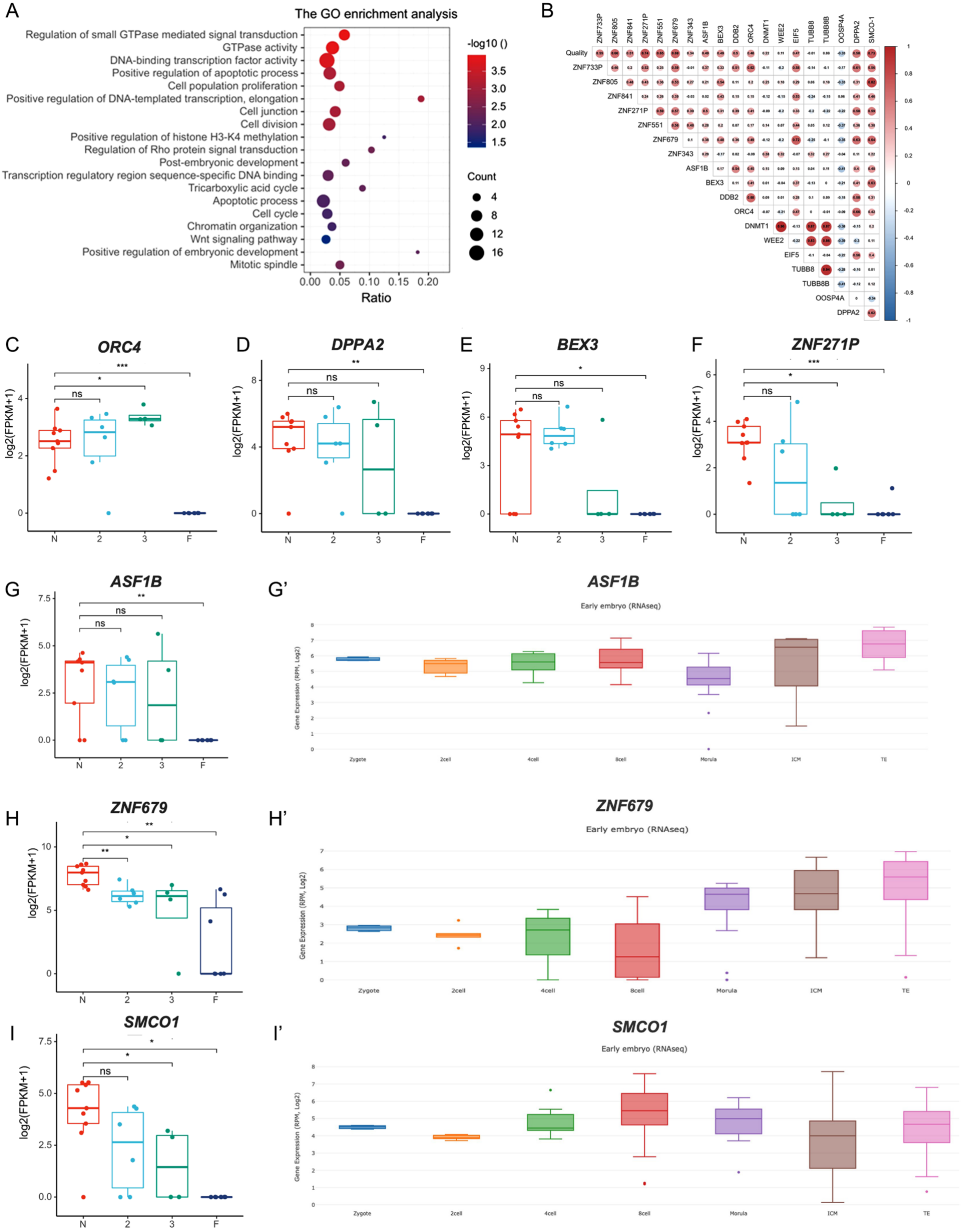
Correlation between gene expressions and embryo developmental competence. (A) Bubble chart shows the enrichment results of gene ontology for all DEGs. (B) The Pearson correlation coefficient values of pairwise comparisons between the selected DEGs and embryonic developmental outcomes. (C-I) The differently expressed *ORC4, DPPA2, BEX3, ZNF271P, ASF1B, ZNF679,* and *SMCO1* among 8-cell embryos in each sample. ns, non-significant. * *P* < 0.05, *** *P <* 0.001. (G’-I’) the expression of *ASF1B, ZNF679,* and *SMCO1* in human early embryos according to the DevOmics database.

The potential role of the DEGs in regulating embryo development was investigated. Firstly, the enrichment gene ontology (GO) for all DEGs was analyzed. As presented in Fig. 6A, GO results suggested that the genes involved in DNA transcription activity, histone methylation, and cell division regulation were highly enriched.

Subsequently, the correlation between DEGs and embryo developmental competence was determined, and the results showed that the expression of a single-pass membrane protein with coiled-coil domains 1 (*SMCO-1*) and Zn finger genes (*ZNF271P* and *ZNF679*) was highly correlated with embryo quality (Fig. 6B). Meanwhile, among these 324 DEGs, the average FPKM value of 65 genes in the 8-cell embryos that failed to form blastocyst was zero. These 65 DEGs were located among 19 autosomes and X chromosome (Supplementary Table 4). The downregulation of seven Zn finger genes (*ZNF733P, ZNF805, ZNF841, ZNF271P, ZNF551, ZNF679,* and *ZNF343*), and maternal eight PRAME family genes was observed in low-quality embryos (Supplementary Table 3). It is also important to notice that the expression of genes such as *ORC4, DPPA2*,* BEX3, ASF1b, DDB2, MDH2, MYG1, UCK1,* and *ZBED5-AS1* was identified in early human embryo according to DevOmics database (Yan *et al.* 2021) and that it plays important roles in regulating embryo development in different animal species. However, the expression of embryo development related genes like *DNMT1, TUBB8B, WEE2,* and* EIF5,* which were identified as key regulators of 8-cell embryo development were not altered.

## Discussion

Following fertilization, the mammalian embryo needs to accomplish the maternal-to-zygotic transition to initiate the development of a new individual. During this period, the defects in embryos or disruptions from the surrounding environment, especially *in vitro* culture conditions, could cause embryo development failure. In the current research, obvious alterations of gene expression in the 8-cell embryos that failed to form blastocysts were detected by evaluating the transcriptions in a single blastomere. The differences in general gene number, cell cycle-related genes, and serval specific genes could serve as markers to predict the developmental potential of human 8-cell embryos.

In ART clinic, the embryos are graded by morphology and cleavage kinetics to determine their quality (Dal Canto *et al.* 2012, Vermeesch *et al.* 2016). Under the specific conditions, preimplantation genetic diagnosis (PGD) might be performed to detect defects in embryos to avoid inheritable diseases (Vermeesch *et al.* 2016). It has been demonstrated that the polar body or cells could be biopsied from the embryo without compromising embryonic viability (He *et al.* 2022). Here, a single blastomere of the 8-cell embryo from the patients who previously experienced embryo development failure due to unknown causes was biopsied for transcriptomic analysis. Compared with the previous study (He *et al.* 2022), our data included samples collected from patients receiving ART for male or tubule factors to serve as a transcriptomic standard for normal embryo development. It was found that the number of transcripts detected in the blastomere of the 8-cell embryos by current scRNA-seq method decreases as their developmental competence decline. Especially the embryos that failed to form blastocysts have significantly fewer transcripts even though their morphology is normal at the 8-cell stage. By further analysis, both maternal and zygotic transcripts were altered in the low-quality embryos indicating that the genomic activation or maternal mRNA degradation process is disrupted at the 8-cell stage, leading to the failure of blastocyst formation. Meanwhile, the cell cycle-related genes also showed abnormal status in low-quality embryos, which is consistent with the result from another research (He *et al.* 2022). Altogether, the global transcriptional alterations in early embryos are tightly associated with their developmental outcomes.

In the current research, the differently expressed genes among the 8-cell embryos were defined as four clusters. First, it was found that the expression of Zn finger genes (*ZNF733P, ZNF805, ZNF841, ZNF271P, ZNF551, ZNF679*, and *ZNF343*) was downregulated in low-quality 8-cell embryos. ZFNs are a family protein playing a critical role in transcriptional regulation, ubiquitin-mediated protein degradation, signal transduction, and other cellular processes (Nakamura *et al.* 2004, Cassandri *et al.* 2017). In the previous study, it has been reported that the expression of *ZNF557* was downregulated in blastocyst formation failure of 8-cell embryos (He *et al.* 2022). The regulatory role of PRAME family proteins in mouse germline development, particularly in the maintenance of embryonic stem cell pluripotency, and the development of primordial germ cells has been identified (Kern *et al.* 2021). In our research, eight PRAME family genes were altered in low-quality human 8-cell embryos, and these genes may be critical in regulating human early embryos development. But the role of ZNFs and PRAME family genes in early embryo has not been elucidated, and functional experiments are needed.

Meanwhile, it was also found that several genes such as *ASF1b, BEX3, DPPA2,* and* ORC4* are correlated with the developmental competence of 8-cell embryos. *ASF1b* is a histone H3–H4 chaperone and plays an essential role in regulating DNA replication, epigenetic modifications, and genome stability in different species (Messiaen *et al.* 2016, Deng *et al.* 2020, Wang *et al.* 2021). Meanwhile, brain expressed X-linked 3 gene (*BEX3*) is identified as an X-linked gene expressed at a high level in preimplantation embryos (Sharov *et al.* 2003). Another gene that was totally suppressed in 8-cell embryos that failed to form blastocyst is developmental pluripotency-associated 2 (*DPPA2*). It has been reported that *Dppa2* and *Dppa4* are positive regulators of mouse 2-cell like cells and transcription of ZGA genes by activating *Dux* (Eckersley-Maslin *et al.* 2019). Recently, another research found that *Dppa2/4* are dispensable for *Dux* and zygotic genome activation in 2C-like state in mouse embryonic stem cells and embryos *in vivo* (Chen *et al.* 2021, Kubinyecz *et al.* 2021), but their maternal stores are critical for offspring survival (Kubinyecz *et al.* 2021). Another noticeable gene is the origin recognition complex subunit 4 (*ORC4*), which is required for DNA replication and exclusion of polar bodies in mouse oocytes (Nguyen *et al.* 2015, 2022). The transcriptional abnormality of those genes could cause the arrest of early embryo development. But further investigation is required to elucidate the underlying molecular mechanism.

## Conclusion

In current research, the transcriptome in single blastomere from human 8-cell embryos was evaluated by scRNA-seq. The altered general gene number and cell cycle-related genes in the 8-cell embryos that failed to form blastocysts were detected. These results could serve as parameters to optimize the embryo culture procedure or PGD method. Furthermore, genes like *SMCO-1, ZNF271P, ZNF679, ASF1b, BEX3, DPPA2,* and* ORC4* are highly correlated with the quality of preimplantation embryos and could be the target for possible rescue therapy methods in the future.

## Supplementary Material

Supplementary Table 1

Supplementary Table 2

Supplementary Table 3

Supplementary Table 4

## Declaration of interest

The authors declare that there is no conflict of interest that could be perceived as prejudicing the impartiality of the research reported.

## Funding

This project was funded by National Key R&D Program of China (2018YFC1003000 and 2020YFA0113200), the National Natural Science Foundation of China http://dx.doi.org/10.13039/501100001809 (32002181 and 31970814), and the 2115 Talent Development Program of China Agricultural University http://dx.doi.org/10.13039/501100002365.
